# Discovery and full genome characterization of a new SIV lineage infecting red-tailed guenons (*Cercopithecus ascanius schmidti*) in Kibale National Park, Uganda

**DOI:** 10.1186/1742-4690-11-55

**Published:** 2014-07-04

**Authors:** Michael Lauck, William M Switzer, Samuel D Sibley, David Hyeroba, Alex Tumukunde, Geoffrey Weny, Anupama Shankar, Justin M Greene, Adam J Ericsen, HaoQiang Zheng, Nelson Ting, Colin A Chapman, Thomas C Friedrich, Tony L Goldberg, David H O’Connor

**Affiliations:** 1 Wisconsin National Primate Research Center, 555 Science Dr, 53705 Madison, WI, USA; 2 Laboratory Branch, Division of HIV/AIDS Prevention, National Center for HIV, Hepatitis, STD, and TB Prevention, Centers for Disease Control and Prevention, Atlanta, GA, USA; 3 Department of Pathobiological Sciences, University of Wisconsin-Madison, Madison, WI, USA; 4 Makerere University, Kampala, Uganda; 5 Department of Anthropology and Institute of Ecology and Evolution, University of Oregon, Eugene, OR, USA; 6 Department of Anthropology and School of Environment, McGill University, Montreal, QC, Canada; 7 Wildlife Conservation Society, Bronx, New York, USA; 8 Department of Pathology and Laboratory Medicine, University of Wisconsin-Madison, Madison, WI, USA

**Keywords:** Simian immunodeficiency virus, SIV, Non-human primates, Guenons, Uganda, Kibale National Park

## Abstract

**Background:**

Human immunodeficiency virus (HIV) type 1 and 2, the causative agents of acquired immunodeficiency syndrome (AIDS), emerged from African non-human primates (NHPs) through zoonotic transmission of simian immunodeficiency viruses (SIV). Among African NHPs, the *Cercopithecus* genus contains the largest number of species known to harbor SIV. However, our understanding of the diversity and evolution of SIVs infecting this genus is limited by incomplete taxonomic and geographic sampling, particularly in East Africa. In this study, we screened blood specimens from red-tailed guenons (*Cercopithecus ascanius schmidti*) from Kibale National Park, Uganda, for the presence of novel SIVs using unbiased deep-sequencing.

**Findings:**

We describe and characterize the first full-length SIV genomes from wild red-tailed guenons in Kibale National Park, Uganda. This new virus, tentatively named SIVrtg_Kib, was detected in five out of twelve animals and is highly divergent from other *Cercopithecus* SIVs as well as from previously identified SIVs infecting red-tailed guenons, thus forming a new SIV lineage.

**Conclusions:**

Our results show that the genetic diversity of SIVs infecting red-tailed guenons is greater than previously appreciated. This diversity could be the result of cross-species transmission between different guenon species or limited gene flow due to geographic separation among guenon populations.

## Findings

Simian immunodeficiency viruses (SIV) naturally infect at least 45 different African non-human primate (NHP) species [1]. Zoonotic transmission of SIVs has led to the emergence of human immunodeficiency virus type 1 (HIV-1) and type 2 (HIV-2) [2,3]. Only African Old World monkeys (OWM) and apes from sub-Saharan Africa, but not their Asian counterparts or New World monkeys, are naturally infected with SIV. The *Cercopithecus* genus, also termed guenons, is of special interest among OWM since it comprises the largest number of species known to harbor SIV [4]. Surprisingly, SIVtal, isolated from a talapoin monkey (*Miopithecus ogouensis*), a different primate genus, also sorts with *Cercopithecus* SIVs, suggesting either evolution from a common ancestor or transmission between genera [5]. Although guenons occupy all of sub-Saharan Africa [6], the vast majority of full-length SIV sequences have been obtained from West and Central African monkeys, potentially influencing our current understanding of the diversity and evolutionary history of *Cercopithecus* SIVs.

Here, we report the discovery and characterization of a novel SIV lineage infecting red-tailed guenons (*Cercopithecus ascanius schmidti*) from Kibale National Park (KNP), the same location where we previously reported SIV in red colobus and black-and-white colobus [7,8]. In 2010, we sampled 12 Kibale red-tailed guenons as part of a larger study of primate ecology, conservation, and health belonging to at least three social groups [9]. All samples were collected within an area of approximately 15 km^2^. Animals were anesthetized and samples were collected as previously described [10]. Plasma samples were then screened for SIV/HIV antibody reactivity using the INNO-LIA HIV-1/2 Score and HIV-2-based Genelabs western blot assays. Viral RNA was prepared from blood plasma for random hexamer-based sequencing as previously described [11]. By deep sequencing, SIV RNA was detected in three out of twelve red-tailed guenons (Table 1). Antibody reactivity was observed in each SIV–positive animal, with the exception of RT03, possibly indicating an acutely infected animal that had not yet mounted an antibody response or insufficient antibody cross-reactivity to the HIV antigens used in serological tests. In two red-tailed guenons (RT04 and RT05), we detected seroreactivity to at least two viral proteins but were unable to recover SIV-specific reads through deep-sequencing (Table 1). We therefore performed RT-PCR, targeting a 400-bp fragment in the C-terminal half of the polymerase (*pol*) gene. A faint product was amplified from both plasma samples and the presence of SIVrtg_Kib was subsequently confirmed by Sanger sequencing (KJ865607- KJ865608). Overall, infection of SIVrtg_Kib in red-tailed guenons was detected in five out of twelve animals, with no restriction to any one social group. This prevalence is comparable to other non-vpu carrying guenons, although the 95% confidence interval of our estimate is wide (19.3% to 68.1%) [4,12-17].

**Table 1:** **Infection of red-tailed (RT) guenons with a novel SIV in Kibale National Park, Uganda**^**1,2**^

**Animal**	**Sex**	**HIV-2 WB**			**HIV-1/2 InnoLIA**			**SIV deep-sequencing**^**3**^**, RT-PCR results**^**4**^
		**Gag**	**Pol**	**Env**	**Gag**	**Pol**	**Env**	
RT01	F	-	-	-	-	-	-	-
RT02	M	-	-	-	-	-	-	-
RT03	F	-	-	-	-	-	-	SIVrtg_Kib + (9473)
RT04	F	w (p26)	-	w (gp80)	-	-	-	-, RT-PCR +
RT05	F	+ (p26)	-	+ (gp80)	1+ (p24)	-	3+ (gp36)	-, RT-PCR +
RT06	M	-	-	-	-	-	-	-
RT08	F	+ (p26)	-	w (gp80)	3+ (p24)	-	3+ (gp120)	SIVrtg_Kib + (1932)
RT09	M	w (p26)	-	-	-	-	-	-
RT10	M	-	-	-	-	-	-	-
RT11	F	-	-	w (gp80)	-	-	-	SIVrtg_Kib + (5618)
RT12	M	-	-	-	-	-	-	-
RT13	M	-	-	-	-	-	-	-

^1^SIV infection was assessed by deep-sequencing of reverse transcribed viral RNA and with serological tests, including HIV-2-specific western blots (HIV-2 WB) and HIV InnoLIA assays.

^2^For HIV-2 WB and HIV-1/-2 InnoLIA, seroreactivity to specific viral proteins is shown in brackets. For the HIV-1/-2 InnoLIA assay, intensity of signals was quantified with "1+" being positive and "3+" being strongly positive. For the HIV-2 WB, intensity of signals was quantified with "w" being weak and "+" being positive.

^3^To quantify the number of SIV-specific reads, read numbers were normalized to 1 million reads per animal.

^4^A 400-bp fragment of the C-terminal half of the polymerase (*pol*) gene was amplified by RT-PCR and the presence of SIVrtg_Kib was subsequently confirmed by Sanger sequencing.

**Figure 1:**
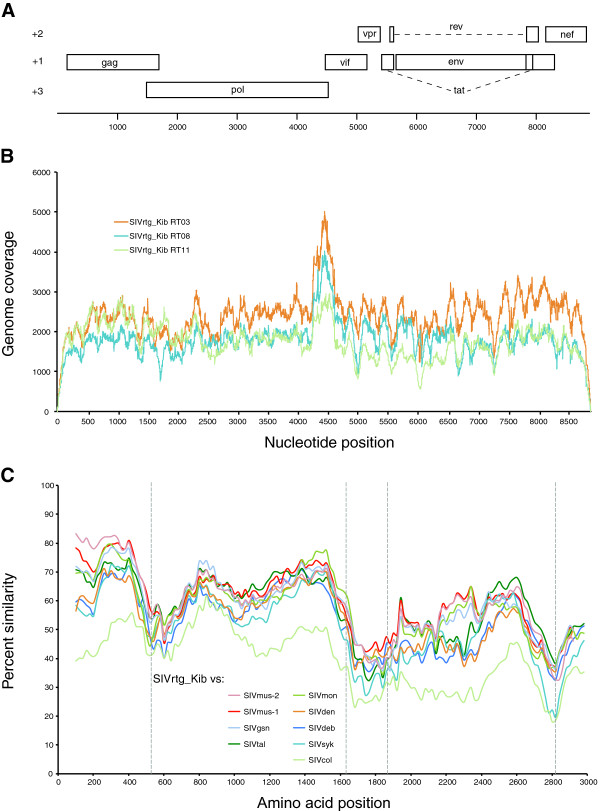
**Genomic organization, deep-sequencing genome coverage and similarity plot analysis of SIVrtg_Kib. A**: Genome organization of SIVrtg_Kib. Boxes represent open reading frames and are drawn to scale in their respective frame. The rev and tat splice variants are indicated by dashed lines. **B**: The SIVrtg_Kib genomes from RT03, RT08 and RT11 were deep-sequenced with four overlapping amplicons. The read coverage at each nucleotide position is shown across the genome. **C**: Sliding window similarity plots of concatenated protein sequences showing the percent similarity of SIVrtg_Kib against other members of *Cercopithecus* SIVs and SIVcol. Dashed vertical lines indicate start positions of viral proteins Gag, polymerase (Pol), Vif, envelope (Env), and Nef.

*De novo* assembly of sequence reads yielded complete SIV coding genomes from three individuals (RT03, RT08 and RT11). The sequence of the *de novo*-assembled genomes was confirmed by deep-sequencing on the Illumina MiSeq, as previously described, resulting in 127,500 – 176,000 reads (average coverage 1750× – 2430×) mapping to the respective genomes (Figure 1B). The five SIVrtg_Kib genomes were highly similar to each other, sharing 96.9 ± 1.1% identity at the nucleotide level (Table 2).

**Table 2:** **Percent nucleotide identity for the five different SIVrtg_Kib isolates**^**1**^

**Nucleotide identity [%]**					
**SIVrtg_Kib**	**03**	**04**	**05**	**08**	**11**
**03**	100				
**04**	99.6	100			
**05**	96.3	96.7	100		
**08**	95.5	95.9	95.1	100	
**11**	97.9	98.4	97.5	96.7	100

^1^The percent nucleotide identity is based on a 243 bp polymerase alignment of the 5 isolates.

A query against the NCBI GenBank database [18] revealed that the new virus was most similar to SIVmus-1, an SIV previously discovered in mustached monkeys (*Cercopithecus cephus*) from Cameroon [12]. This finding was confirmed by pairwise alignment of the coding regions of SIVrtg_Kib and representatives from major SIV lineages (Table 3). The genomic structure of SIVrtg_Kib is similar to that of other SIVs, including all three structural genes (*gag*, *pol* and *env*) as well as accessory genes (*vif*, *vpr*, *tat*, *rev* and *nef*) (Figure 1A). Several *Cercopithecus* SIVs are characterized by the presence of a *vpu* gene (SIVmus, SIVmon, SIVgsn, SIVden). However, like SIVdeb, SIVsyk and SIVtal, SIVrtg_Kib does not encode a *vpu* homolog. Functional motifs in Env and Gag resemble those of other *Cercopithecus* SIVs, containing 18 cysteine residues in the extracellular glycoprotein gp120 as well as both the PT/SAP and YPXL budding motifs in the Gag protein [19].

**Table 3:** Percent nucleotide identity of concatenated Gag-Pol-Env-Nef sequences along the coding region for SIVrtg_Kib and other major SIV lineages

**Nucleotide identity [%]**													
**SIV strains**	**rtg_Kib**	**mus-1**	**mus-2**	**deb**	**tal**	**cpz**	**syk**	**mnd-2**	**agm**	**rcm**	**smm**	**lst**	**col**
**rtg_Kib**	100												
**mus-1**	63.2	100											
**mus-2**	62.6	75.1	100										
**deb**	55.1	55.3	54.9	100									
**tal**	57.5	56.6	56.1	57.8	100								
**cpz**	56.1	56.4	56.5	52.5	52.3	100							
**syk**	54.4	53.1	54.3	59.8	56.1	51.1	100						
**mnd-2**	52.3	52.0	52.3	54.0	54.6	54.3	53.0	100					
**agm**	53.6	51.9	52.5	53.8	55.6	52.5	54.5	57.6	100				
**rcm**	52.7	51.7	51.7	53.8	54.8	54.5	54.0	63.9	59.0	100			
**smm**	52.8	52.7	53.1	55.1	55.5	52.7	54.0	59.2	58.4	61.4	100		
**lst**	50.8	50.0	50.1	52.1	52.4	50.3	51.9	57.0	55.1	55.5	55.1	100	
**col**	47.8	48.1	47.4	48.5	47.6	47.9	47.0	49.4	49.3	48.9	48.8	48.8	100

**Figure 2:**
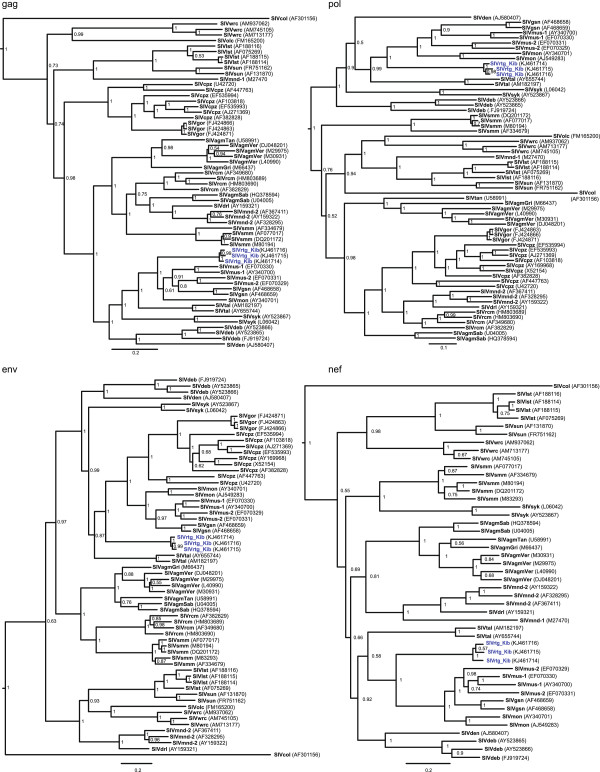
**Phylogenetic relationship of newly discovered SIVrtg_Kib to other SIVs.** Separate Bayesian Markov Chain Monte Carlo phylogenies were constructed for *gag*, polymerase (*pol*), envelope (*env*) and *nef* proteins. Posterior clade probabilities are shown at branch nodes. The scale bar below the phylogenetic trees represents substitutions per site. The newly discovered SIVrtg_Kib is highlighted in blue.

We analyzed the amino-acid similarity of the novel SIV with related SIV lineages and SIVcol across Gag, Pol, Vif, Env and Nef using SimPlot v3.5.1 [20], following TranslatorX alignment (MAAFT) [21]. Based on the SimPlot analysis, SIVrtg_Kib seems to be equidistantly related to the other members of the *Cercopithecus* genus across Pol, Vif, Env and Nef, while the Gag protein shares the highest sequence identity with SIVs isolated from mustached monkeys (SIVmus-1 and SIVmus-2) (Figure 1C). We also estimated the phylogenetic position of SIVrtg_Kib using SIV lineages with complete genomes. Briefly, nucleotide sequences of *gag*, *pol*, *env* and *nef* were codon aligned individually using ClustalW and edited manually, followed by Bayesian analysis using the BEAST v1.6.2 program [22]. The Bayesian phylogeny shows that across all four genes examined, SIVrtg_Kib forms a separate lineage that clusters with the other *Cercopithecus*-specific SIVs and SIVtal (Figure 2). Within the *Cercopithecus* SIV group, SIVrtg_Kib is ancestral to the SIVgsn/mon/mus lineage in each gene with strong support, which includes SIVs from greater spot-nosed guenons (SIVgsn; *C. nictitans*), mona monkeys (SIVmon; *C. mona*) and mustached monkeys (SIVmus-1 and SIVmus-2).

**Figure 3:**
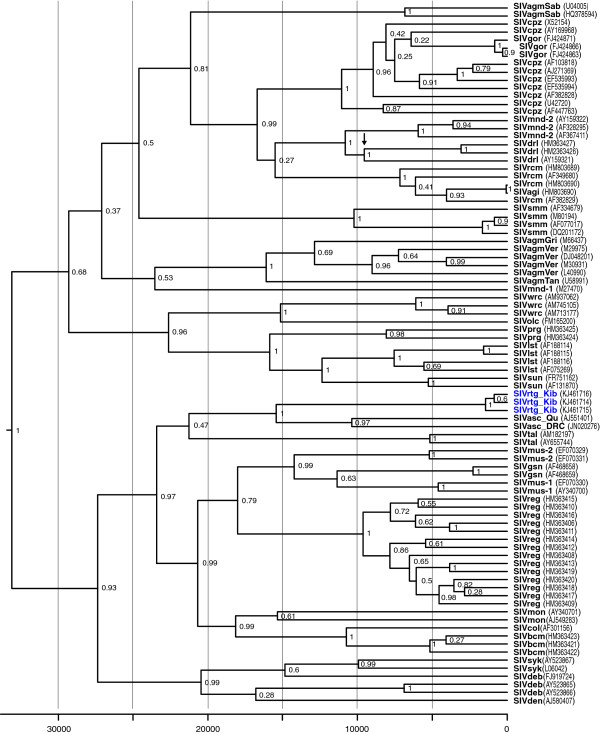
**Time to most recent common ancestor (TMRCA) for SIVrtg_Kib and other representative SIVs.** Bayesian Markov Chain Monte Carlo phylogenies were generated in order to estimate TMRCA by calibrating the relaxed molecular clock using the estimated 10,000 year old separation of the drill (*Mandrill leucophaeus*) SIVs on mainland Africa from those on Bioko Island, Equatorial Guinea [25]. The scale bar below the phylogenies represents years before present and the black arrow represents the Bioko calibration point used in this analysis. Posterior clade probabilities are shown at branch nodes. The newly discovered SIVrtg_Kib is highlighted in blue.

In 2004, Verschoor *et al*. described the discovery of SIVasc_Qu from a red-tailed guenon in a zoo in the Netherlands [23]. This virus was isolated from the same subspecies in which we discovered SIVrtg_Kib in Kibale National Park, however the authors were only able to recover a 1895-bp partial *pol* sequence. In 2011, Ahuka-Mundeke and colleagues discovered a second SIV from red-tailed guenons in the Democratic Republic of Congo (SIVasc_DRC), although from a different sub-species, *Cercopithecus ascanius whitesidei*[13]. Ahuka-Mundeke *et al.* were able to only recover a 648-bp partial *pol* fragment, probably due to limitations associated with nucleic acid recovery from bushmeat. Because full genomes were unavailable for both viruses, we did not include them in our sliding window similarity plot analysis. We did, however, determine the pairwise genetic distance of SIVrtg_Kib to SIVasc_Qu and other *Cercopithecus* SIVs by aligning corresponding sequences to the 1895 nt partial *pol* sequence initially recovered from SIVasc_Qu. Our new SIVrtg_Kib is as divergent from SIVasc_Qu as from the other *Cercopithecus* SIVs, sharing 66% identity with SIVasc while sharing 65.6 ± 1.8% identity with the remaining SIVs in this genus. The high degree of sequence divergence could be a result of the vast geographical range of *C. ascanius schmidti*, stretching from the Congo-Oubangui River system in central Africa, through Uganda to the Rift Valley in Kenya and western Tanzania [24]. Similarly, a pairwise comparison of SIVasc_DRC to SIVrtg_Kib revealed that both viruses were equally divergent, sharing 69.8% nucleotide identity. We also performed a separate phylogenetic analysis that included all three SIVs isolated from red-tailed guenons as well as other representative SIV lineages and determined the time to most recent common ancestor (TMRCA) using Bayesian inference and calibration of the molecular clock using an the estimated 10,000 year old separation of the drill (*Mandrill leucophaeus*) SIVs on mainland Africa from those on Bioko Island, Equatorial Guinea, as previously described [8]. The root of the tree is estimated to be 33,394 years before present (ybp) (95% highest posterior density (HPD) = 19,157 – 51,174 ybp) and is thus comparable to that inferred for the Bioko monkey SIV phylogenies (49,129 ybp; 95% HPD = 29,078 - 71,268 ybp) [25] (Figure 3). While SIVrtg_Kib groups together with SIVasc_Qu and SIVasc_DRC, there is a split between SIVrtg_Kib and SIVasc_Qu/DRC that occurred at least 15,500 ybp (95% HPD = 7554 – 15,883 ybp). We also acknowledge that despite the use of a strong geological calibration point for our molecular dating estimates, considerable debate exists about the accuracy of SIV TMRCA estimates and suggest that dates should be regarded as minimum estimates. Evidence of genetic recombination in the SIVrtg-Kib was not observed in either bootscan analysis using the Simplot program (data not shown) or in the topologies inferred for each of the major gene regions and thus recombination likely did not influence the phylogenetic analyses.

In this study, we describe the first full-length SIV genome sequence isolated from the blood plasma of wild-caught red-tailed guenons. The high diversity observed among SIVs isolated from this species, as well as from other *Cercopithecus* SIVs from West and Central Africa, could potentially be explained by geographic separation of host species. Guenons are distributed throughout most of Sub-Saharan Africa and live in populations separated by large distances or geographic barriers [6]. As a consequence, limited contact between red-tailed guenons from different areas of Africa could limit the transmission of SIVs among guenon populations. Alternatively, cross-species transmission among different guenon taxa may have contributed to SIV diversity, which would not be surprising given that sympatric guenons are known to hybridize in both wild and captive settings. The complex evolutionary relationships among taxa within the genus *Cercopithecus* suggest that additional sampling of the guenons may be fruitful for understanding the co-evolution of SIVs and their hosts.

## Competing interests

The authors declare that they have no competing interests.

## Authors’ contributions

Conceived and designed the experiments: DHO TLG TCF NT CAC WMS ML. Performed the experiments: ML SDS WMS AS HZ. Analyzed the data: ML WMS JMG AJE DHO. Wrote the paper: ML WMS TLG DHO TCF. Conducted study in the field: DH AT GW TLG. All authors read and approved the final manuscript.
